# *Clonorchis sinensis* aggravates biliary fibrosis through promoting IL-6 production via toll-like receptor 2-mediated AKT and p38 signal pathways

**DOI:** 10.1371/journal.pntd.0011062

**Published:** 2023-01-24

**Authors:** Yuru Wang, Xu Zhang, Xiaocen Wang, Nan Zhang, Yanhui Yu, Pengtao Gong, Xichen Zhang, Yeting Ma, Xin Li, Jianhua Li

**Affiliations:** Key Laboratory of Zoonosis Research, Ministry of Education; College of Veterinary Medicine, Jilin University, Changchun, China; Baylor College of Medicine, UNITED STATES

## Abstract

*Clonorchis sinensis* is an important food-borne zoonotic parasite which has been linked to biliary fibrosis and cholangiocarcinoma. However, the details of the pathogenesis of *C*. *sinensis* were unclear. To explore the role and regulatory mechanism of toll-like receptor 2 (TLR2) in *C*. *sinensis-*induced biliary fibrosis, we established the *C*. *sinensis*-infected C57BL/6 mouse model with *TLR2^-/-^* and wild type (WT) mice. The mortality rate, liver lesions, TLR2 and TGF-β1 expression, phosphorylation of Smad2/3, AKT, p38, ERK and p65, and cytokine productions were analyzed. Furthermore, similar parameters were examined in mouse biliary epithelial cells (BECs) co-cultured with *C*. *sinensis* excretory/secretory proteins (ESPs). The results showed that TLR2 expression was enhanced significantly in *C*. *sinensis*-infected WT mice and mouse BECs. *C*. *sinensis*-infected *TLR2^-/-^* mice exhibited an increased weight and a decreased mortality rate; significantly alleviated liver lesions and biliary fibrosis, reduced numbers of myofibroblasts; decreased expression of TGF-β1 and phosphorylation level of AKT, p38 and Smad2/3; significantly decreased production of IL-6, TNF-α and IL-4, while increased production of IFN-γ compared with *C*. *sinensis*-infected WT mice. Furthermore, *C*. *sinensis* ESPs could activate TLR2-mediated AKT and p38 pathways to increase the production of IL-6 in mouse BECs. In conclusion, these data indicate that *C*. *sinensis* infection activated TGF-β1-Smad2/3 through TLR2-mediated AKT and p38 pathways to promote IL-6 production, which resulted in myofibroblast activation and aggravating biliary fibrosis in mice.

## Author summary

*Clonorchis sinensis* is one of the most prevalent and harmful food-borne parasitic diseases in Asian countries and is closely associated with cholangitis, cholecystitis, biliary fibrosis and cholangiocarcinoma. However, the pathogenesis of biliary fibrosis caused by *C*.*sinensis* has not been fully elucidated yet. In this study, we established a liver fibrosis model using TLR2-deficient mice and TLR2-normal mice to investigate the role and regulatory pathways of TLR2 in regulating biliary fibrosis induced by *C*. *sinensis*. A new role of TLR2 in aggravating *C*. *sinensis*-induced parasitic liver fibrosis was identified. *C*. *sinensis* ESPs activated TLR2-mediated AKT and p38 pathways to increase the production of IL-6 in mouse BECs *in vitro*. This result supported the finding that TLR2 triggered TGF-β1/Smad2/3 via promoting the production of IL-6 played crucial roles in activating myofibroblasts, which exacerbated liver fibrosis caused by *C*. *sinensis*. This study provides a useful mouse model to study the potential mechanisms of parasite-induced liver fibrosis, and explores the role of TLR2 deficiency in liver fibrosis, providing important reference information for understanding liver fibrosis caused by *C*. *sinensis*.

## Introduction

*Clonorchis sinensis*, an important food-borne zoonotic parasite, has been linked to liver fibrosis and cholangiocarcinoma [1]. About 15 million people are infected by this liver fluke in east Asia including China. *C*. *sinensis* has become one of the most prevalent and harmful food-borne parasitic diseases in China [1]. The bile ducts are the normal parasitic site of *C*. *sinensis*. However, the immune response of BECs during *C*. *sinensis* infection has not been extensively investigated. In the acute infection of *C*. *sinensis*, nausea, abdominal pain, diarrhea, and eosinophilia can occur, while long-term infection may lead to gall stones, cholangitis, biliary fibrosis, liver cirrhosis and even cholangiocarcinoma and hepatocellular carcinoma [1]. Previous studies suggested that the incidence of liver cirrhosis in *C*. *sinensis* patients is 6 times higher than those not infected [2]. *C*. *sinensis* is regarded as a Class I carcinogen by the International Agency for Research on Cancer (IARC) [3] and is also listed as an important pathogen of neglected tropical disease [4]. Praziquantel can be used as a potent anthelmintic. To date no effective vaccine is available for clonorchiasis prevention [1]. Understanding of liver fibrosis and knowledge of these pathways may be beneficial for clinical care, and broaden prevention and control approaches.

Liver fibrosis caused by *C*. *sinensis* is characterized by bile duct hyperplasia of epithelial cell damage, inflammatory cell infiltration in the hepatic portal area [5] and extracellular matrix (ECM) massive deposition around the bile duct [6]. C3H/HeN, C57BL/6, BALB/c, ICR, DDY and CBA/N mice were used to establish *C*. *sinensis*-infected mouse model [7]. TLR4 was highly expressed in the liver of *C*. *sinensis*-infected C3H/HeN mice and exacerbated biliary fibrosis by activating hepatic stellate cells (HSCs) through the TLR4-p65-TGF-β1/Smads pathway [6].

TLR2, as an important innate immune receptor, can sense both endogenous and exogenous ligands to initiate danger signals and to induce inflammatory responses [8]. It has been shown that TLR2 was significantly increased in *C*. *sinensis*-infected mouse model [9]. Recombinant *Clonorchis sinensis* HscB (rCsHscB) from *C*. *sinensis* interacts with TLR2 to promoted the expression of IL-10 in mouse macrophage [10]. Modulation of TLR2 function affected inflammatory process in chronic hepatitis (B, C), nonalcoholic fatty liver disease and alcoholic liver disease [8,11,12]. Regulation of TLR2 function affects the inflammatory process in alcoholic liver disease, viral liver disease and other liver diseases. As a result, TLR2 has been considered as the potential target for the treatment of liver fibrosis [13,14]. Further study confirmed that TLR2 was present in human biliary epithelial cells and involved in the innate immunity of the intrahepatic biliary tree [15]. The role and mechanism of TLR2 in biliary fibrosis induced by *C*. *sinensis* remain unclear. The biliary fibrosis mouse model induced by *C*. *sinensis* infection is essential, which will help to explore the role of TLR2 and the changes of pathways regulated by TLR2 during *C*. *sinensis* infection.

The *C*. *sinensis*-induced biliary fibrosis mouse model was established using TLR2-deficient or WT C57/BL6 mice. We examined mouse mortality rate, weight loss, TLR2 signal pathways, cytokine production, as well as liver and bile duct lesions in *C*. *sinensis*-infected *TLR2^-/-^* and WT mice. Furthermore, we explored the mechanisms of TLR2-regulated cytokines expression in *C*. *sinensis* ESPs-stimulated BECs.

## Materials and methods

### Ethics statement

All animal experimental procedures were in accordance with Chinese legal standards and the conduct of the experiments was approved by the Animal Welfare and Research Ethics Committee at Jilin University (IACUC Permit Number: 20160612).

### Animals

*TLR2^-/-^* C57BL/6 mice were purchased from the Model Animal Research Center of Nanjing University, and WT female C57BL/6 mice were obtained from the Beijing HFK Bioscience Co. Ltd. All mice were housed under constant temperature, pathogen-free animal conditions for 12 h on a dark/light cycle. The sterile normal mouse chow and water were provided to the mice.

### Isolation of mouse BECs

The isolation and culture of BECs followed the previously described methods [16]. Briefly, WT and *TLR2^-/-^* mice (female, 6-8 weeks) bile ducts were stripped and cut into tiny pieces at aseptically. Tissue pieces were shaken and digested for 30 min at 37°C in 0.5% collagenase IV (Sigma-Aldrich, St. Louis, USA), then were differential density gradient centrifugation and were filtered through a 74μm/aperture metal sieve. 3 ×10^5^ cells were plated in a well of 6-well tissue culture plates. Cells were washed thrice with sterilized phosphate buffer saline (PBS) (pH 7.4) before co-incubation to remove cell debris and residual medium.

### *C*. *sinensis* metacercariae and ESPs collection

*C*. *sinensis* metacercariae were isolated from *Pseudorasbora parva*, the second intermediate host, which were collected from the *C*. *sinensis* endemic area [17]. *C*. *sinensis* ESPs were prepared as previously described [18]. Adults of *C*. *sinensis* were isolated from metacercariae infected WT mice and incubated at 37°C for 3 h at density of five parasites per mL of serum-free RPMI-1640 containing a mixture of antibiotics [18].

### *C*. *sinensis*-infected mouse model

WT mice were divided into 6 groups with 10 mice per group. The mice were inoculated with 50, 100, 200, 400, 800 metacercariae by gavage in 200 μL sterile PBS (pH 7.4), and mice given 200 μL sterile PBS were used as the control. The mortality of mice was recorded daily and mice were euthanized at 7 days post infection (dpi), 15dpi, and 35dpi, respectively. The intrahepatic parasites were collected and counted. Livers were isolated and used for cytokines detection, Western blot, histological observation, Masson staining and immunohistochemical staining.

### Real-Time PCR analysis

Total RNA of biliary epithelial cells was extracted using the Trizol reagent (Invitrogen, Carlsbad, CA, USA) and was used for Quantitative Real-Time PCR (qPCR) [19]. The primer sequences were TLR2-F 5’-CCCACTTCAGGCTCTTTGAC-3’, TLR2-R 5’-GCCACTCCAGGTAGGTCTTG-3’ and β-actin-F 5’-TGCTGTCCCTGTATGCCTCT-3’, β-actin-R 5’-GGTCTTTACGGATGTCAACG-3’ [20].

### Cytokines detection by ELISA

3×10^5^/well *TLR2^-/-^* and WT mouse BECs were plated in a well of six-well tissue culture plates and incubated for 18 h with or without 50 μg/mL *C*. *sinensis* ESPs. The BECs added with 1640 were as control group. For kinase inhibition experiments, BECs were pretreated with inhibitors of AKT (5 μM) (Santa Cruz), p38 (30 μM) or ERK (40μM) (Sigma-Aldrich, St. Louis, USA) for 60 min at 37°C before treatment with *C*. *sinensis* ESPs [19]. Following the manufacturer’s instructions, the productions of IL-6, TGF-β1, TNF-α, IL-4 and IFN-γ were detected with ELISA Kit (Thermo Scientific, Massachusetts, USA) [19].

### Histological observation

At the time of sacrifice, 2 g of liver tissues were sliced with the thickness of 3 μm then routine deparaffinization was performed with xylene and stained with hematoxylin-eosin (H&E) staining. Hepatic injures and inflammation were faithfully documented under the microscope, and the hepatic inflammation in mice was evaluated using hepatic histological activity index.

### Masson staining

Masson trichrome staining was used to visualize liver fibrosis. The tissue section was stained with the Masson’s Trichrome Stain Kit (Solarbio, Beijing, China) according to the manufacturer’s recommendations. The collagen deposition area was digitized and quantified by Image-Pro Plus software.

### Immunohistochemical staining

The tissues were sliced with the thickness of 5 μm sections with routine deparaffinization. The sections were incubated with rabbit-anti-TLR2 antibody, rabbit-anti-α-SMA, rabbit-anti-CK-19 (Abcam, Cambridge, USA), Goat-anti-rabbit IgG H&L (HRP) (Affinity bioscience, Jiangsu, China) and DAB staining (Solarbio, Beijing, China). Details of the antibodies were presented in Table 1. The expression levels of TLR2 in liver were digitized and analyzed using Image-Pro Plus software.

**Table 1:** Details of the antibodies used in this study.

Antibodies	Description	Isotype	Dilution ratio	manufacturer
**p65**	monoclonal	Rabbit IgG	1:1000	Cell Signaling Technology (Shanghai, China)
**Phospho-p65**	Rabbit monoclonal	Rabbit IgG	1:1000	Cell Signaling Technology
**AKT**	Rabbit monoclonal	Rabbit IgG	1:1000	Cell Signaling Technology
**Phospho-AKT**	Rabbit monoclonal	Rabbit IgG	1:1000	Cell Signaling Technology
**Smad2/3**	Rabbit polyclonal	Rabbit IgG	1:1000	Abcam (Cambridge, USA)
**Phospho-Smad2/3**	Rabbit polyclonal	Rabbit IgG	1:1000	Abcam
**P38**	Rabbit monoclonal	Rabbit IgG	1:1000	Cell Signaling Technology
**Phospho-p38**	Rabbit monoclonal	Rabbit IgG	1:1000	Cell Signaling Technology
**ERK**	Rabbit monoclonal	Rabbit IgG	1:1000	Cell Signaling Technology
**Phospho-ERK**	Rabbit monoclonal	Rabbit IgG	1:1000	Cell Signaling Technology
**GAPDH**	Rabbit monoclonal	Rabbit IgG	1:1000	Cell Signaling Technology
**TLR2**	Rabbit monoclonal	Rabbit IgG	1:100	Abcam
**α-SMA**	Rabbit monoclonal	Rabbit IgG	1:400	Abcam
**CK-19**	Rabbit monoclonal	Rabbit IgG	1:400	Abcam
**HRP-link-antibodies**		Goat-anti- rabbit IgG	1:5000	Affinity bioscience (Jiangsu, China)

### Western blot analysis

The liver tissues of mice were made into single cells. The WT and *TLR2^-/-^* mouse BECs were co-cultured with *C*. *sinensis* ESPs (50 μg/mL) for 120 min. SDS-PAGE and membrane transfer tests were performed in accordance with the previous method. The membranes were incubated with primary antibodies at 4°C overnight. After all membranes were washed with PBST, incubated with HRP-conjugated antibody at room temperature for 1 h. The membranes were washed three times with PBST and then data were collected using Clinx ChemiScope Series (Clinx Science Instrument Co., Ltd, Shanghai, China). Details of the antibodies were presented in Table 1.

### Statistical analysis

GraphPad Prism 6.01 (GraphPad Software, Inc., California, USA) was used to conduct one-way ANOVA and Tukey test on the data and generate pictures. The data were expressed as the mean ± SD of three independent experiments. Significance was set at *p < 0.05, **p < 0.01, and ***p < 0.001.

## Results

### 1. High TLR2 expression in mouse BECs caused by *C*. *sinensis*

**Fig 1:**
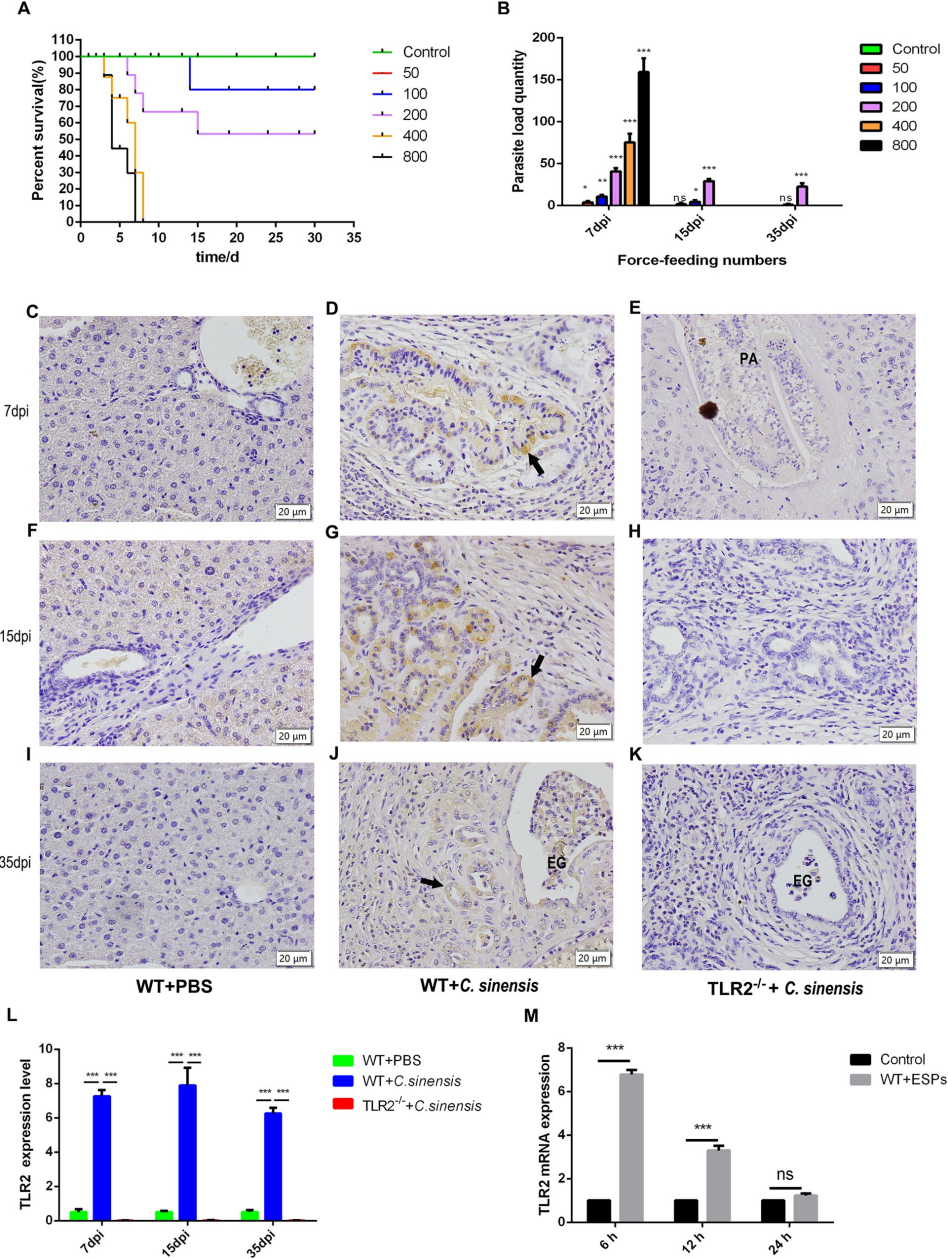
*C*. *sinensis* induced prominently expressed TLR2 in mouse BECs. (A, B) The survival rate and intrahepatic parasites for mice infected with difference *C*. *sinensis* metacercariae. WT mice (n = 10 mice per group) were orally infected with 0, 50, 100, 200, 400, 800 *C*. *sinensis* metacercariae, respectively. (A) The dead mice were recorded daily, and the survival curve was depicted. (B) Mice infected with *C*. *sinensis* were euthanized and the parasites from the livers were collected and calculated at 7 dpi, 15 dpi and 35 dpi. (C-L) TLR2 expression in mouse BECs. The WT and *TLR2*^*-/-*^ mice were orally administrated by 200 *C*. *sinensis* metacercariae or PBS, respectively. These mice were euthanized and livers were harvested at 7 dpi, 15 dpi and 35 dpi. The liver tissues were sliced into 5 μm and stained with antibodies against TLR2. The black arrow indicated TLR2 expression in BECs. Eggs were indicated by ‘EG’; Parasites were indicated by the letter ‘PA’. Scale bars = 20 μm. (J) The mean optical density of TLR2 expression indicated by immunohistochemical staining was digitized and quantitated by Image-Pro Plus software. (M) 3 × 10^5^ mouse BECs were stimulated with *C*. *sinensis* ESPs (50 μg/mL) at 6 h, 12 h and 24 h. TLR2 mRNA expression levels were detected by qPCR analysis. The mRNA level was normalized to β-actin.

The results of mortality of *C*. *sinensis*-infected mice showed that all mice infected with 400/800 metacercariae died at the early stage of infection (<10 d). The infection rate of mice infected with 200 metacercariae was 100% and the average mortality of them was 40.00%. Mice infected with 100 metacercariae had an average infection rate of 23.33% and a mortality of 10.00%. The average infection rate of mice infected with 50 metacercariae was 6.67% (Fig 1A). The intrahepatic parasites of mice infected with *C*. *sinensis* metacercariae (50, 100, 200) at 35dpi were 0.67, 2.22, 22.67 (Fig 1B). Based on the mortality and the intrahepatic parasites of mice, the number of metacercarias in infected mice was determined as 200 metacercarias per mouse.

Then we established a *C*. *sinensis*-infected mouse model in *TLR2^-/-^* and WT mice. Immunohistochemistry results showed that TLR2 expression level in BECs of *C*. *sinensis*-infected WT mice was significantly increased at 7 dpi, 15 dpi and 35 dpi compared to that in PBS control group (Fig 1C–1L) (*p*<0.001, *p*<0.001, *p*<0.001), while TLR2 was not detected in *TLR2^-/-^* mice (Fig 1C–1L) (*p*<0.001, *p*<0.001, *p*<0.001). In addition, qPCR results revealed that TLR2 mRNA expression in BECs of WT mouse incubated with *C*. *sinensis* ESPs exhibited a significant increase at 6 h, compared with control without stimulation (Fig 1M) (*p*<0.001). The results showed that *C*. *sinensis* promoted TLR2 expression in mouse BECs both *in vivo* and *in vitro*.

### 2. *TLR2^-/-^* mice showed decreased mortality rate, reduced weight loss after *C*. *sinensis* infection

**Fig 2:**
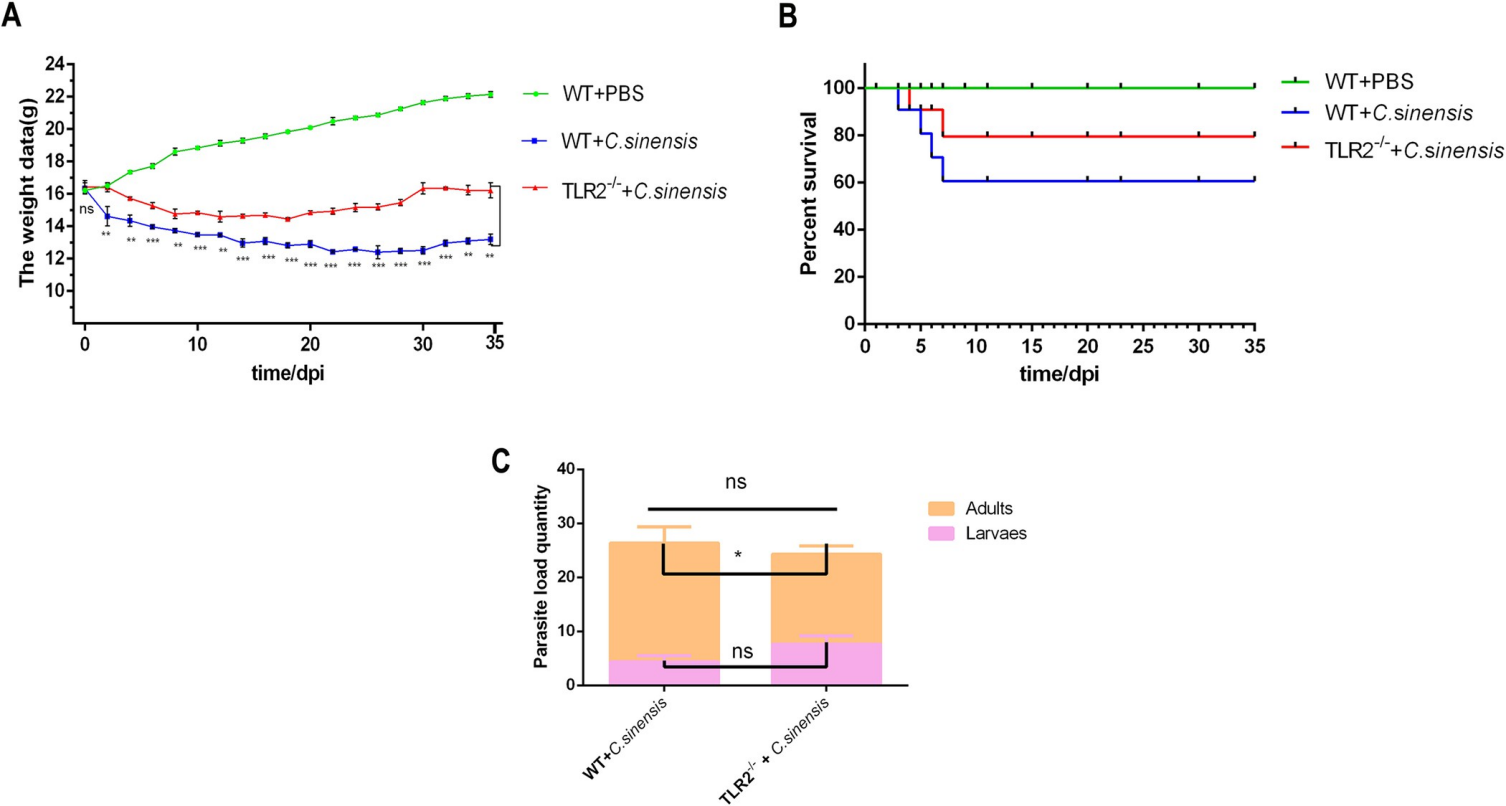
*TLR2*^*-/-*^ mice decreased mortality rate, weight loss caused by *C*. *sinensis*. The WT and *TLR2*^*-/-*^ mice were orally administrated by 200 *C*. *sinensis* metacercariae or PBS, respectively. (A) Mice were weighed every day and weight change curve was drawn. (B) The dead mice were recorded daily, and the survival curve was depicted. (C) The intrahepatic parasites were collected from *C*. *sinensis*-infected WT and *TLR2*^*-/-*^ mice at 35 dpi. *C*. *sinensis* adults and juvenile flukes were distinguished and counted. The data were expressed as the mean ± SD of three independent experiments.

The weight of *C*. *sinensis*-infected WT mice was significantly decreased (Fig 2A), and mice began to die at 3 dpi, with an overall mortality rate of 40% (Fig 2B). Compared with *C*. *sinensis*-infected WT mice, *C*. *sinensis*-infected *TLR2^-/-^* mice showed reduced weight loss (Fig 2A) (*p*<0.01) with an average mortality rate at 20% (Fig 2B). The present data indicated that TLR2 deficiency improved survival performance caused by *C*. *sinensis* in mice. The number of intrahepatic parasites in *C*. *sinensis*-infected *TLR2^-/-^* and WT mice were statistically analyzed Although no difference in total numbers of intrahepatic parasites was observed between the two groups, there was a statistically significant reduction in those reaching adult stage in *TLR2^-/-^* mice than that in WT mice (Fig 2C).

### 3. TLR2 deficiency reduced bile duct lesions and liver inflammation in *C*. *sinensis*-infected mice

**Fig 3:**
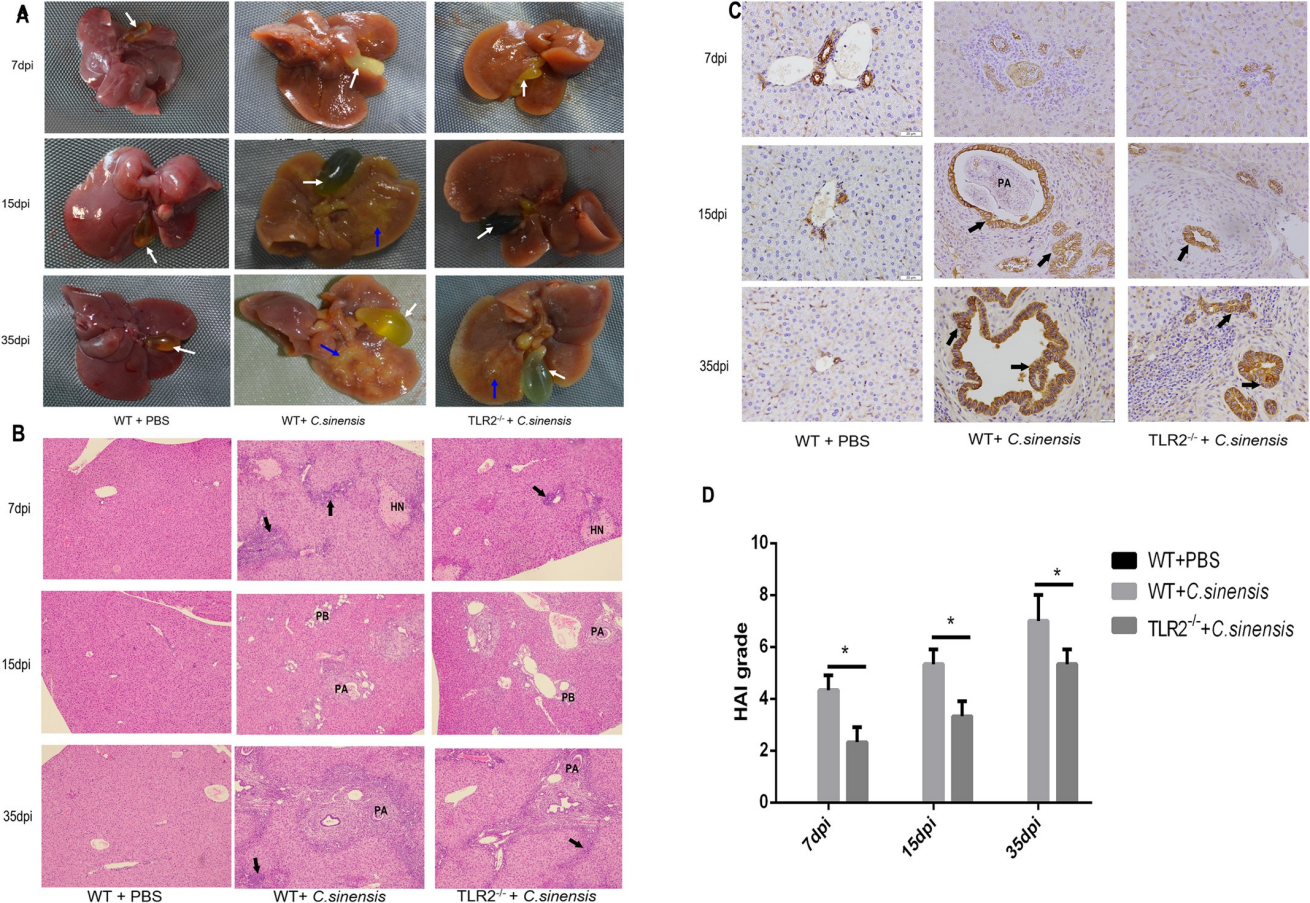
*C*. *sinensis* enhanced bile duct and liver injury through TLR2 pathway. (A) Liver lesions of *C*. *sinensis*-infected WT and *TLR2*^*-/-*^ mice at 7dpi, 15dpi and 35 dpi were observed. *C*. *sinensis* caused hepatomegaly, jaundice, cholestasis, connective tissue hyperplasia, and bile ducts dilated protrusions, white arrow pointed to gallbladder, and blue arrow pointed to bile ducts dilated protrusions. (B) Liver tissue sections were prepared and stained with H&E. Histopathological changes of *C*. *sinensis*-infected WT and *TLR2*^*-/-*^ mice at different time points were observed. The oozed inflammatory cells were indicated by the black arrows. Hepatic cells necrotic was indicated by ‘HN’; Obstruction of the biliary tract by parasites was indicated by the letter ‘PA’. Scale bars = 50 μm. (C) Immunohistochemistry for CK-19 in the liver from WT and *TLR2*^*-/-*^ mice at 7 dpi, 15 dpi and 35 dpi of *C*. *sinensis* infection. The BECs hyperplasia were indicated by the black arrows. Obstruction of the biliary tract by parasites was indicated by the letter ‘PA’. Scale bars = 20 μm. (D) The degree of liver inflammation and injury was calculated by hepatic HAI.

Hepatomegaly, jaundice, cholestasis and dilated protrusions of bile ducts were found in *C*. *sinensis-*infected WT mice. TLR2 deficiency reduced liver connective tissue hyperplasia and bile duct lesions caused by *C*. *sinensis* compared to WT mice (Fig 3A). In PBS control mice, the BECs showed structural integrity and normal lumen morphology (Fig 3B and 3C). For *C*. *sinensis* infected WT mice, there were obvious inflammatory cells infiltration, hepatocellular necrosis foci and obstruction of the biliary tract by parasites (Fig 3B). *C*. *sinensis* caused cholangiectasis, bile duct epithelial hyperplasia, deformation and epithelial ulceration (Fig 3C). The histological activity index (HAI) of *C*. *sinensis*-infected WT mice continued to increase, and the inflammation rating of *C*. *sinensis*-infected *TLR2^-/-^* mice was significantly lower than that in WT mice (Fig 3D) (*p*<0.05, *p*<0.05, *p*<0.05). These data indicated that TLR2 deficiency reduced the inflammatory and injury of bile ducts and liver caused by *C*. *sinensis* in mice.

### 4. TLR2 deficiency alleviated biliary fibrosis induced by *C*. *sinensis* in mice

**Fig 4:**
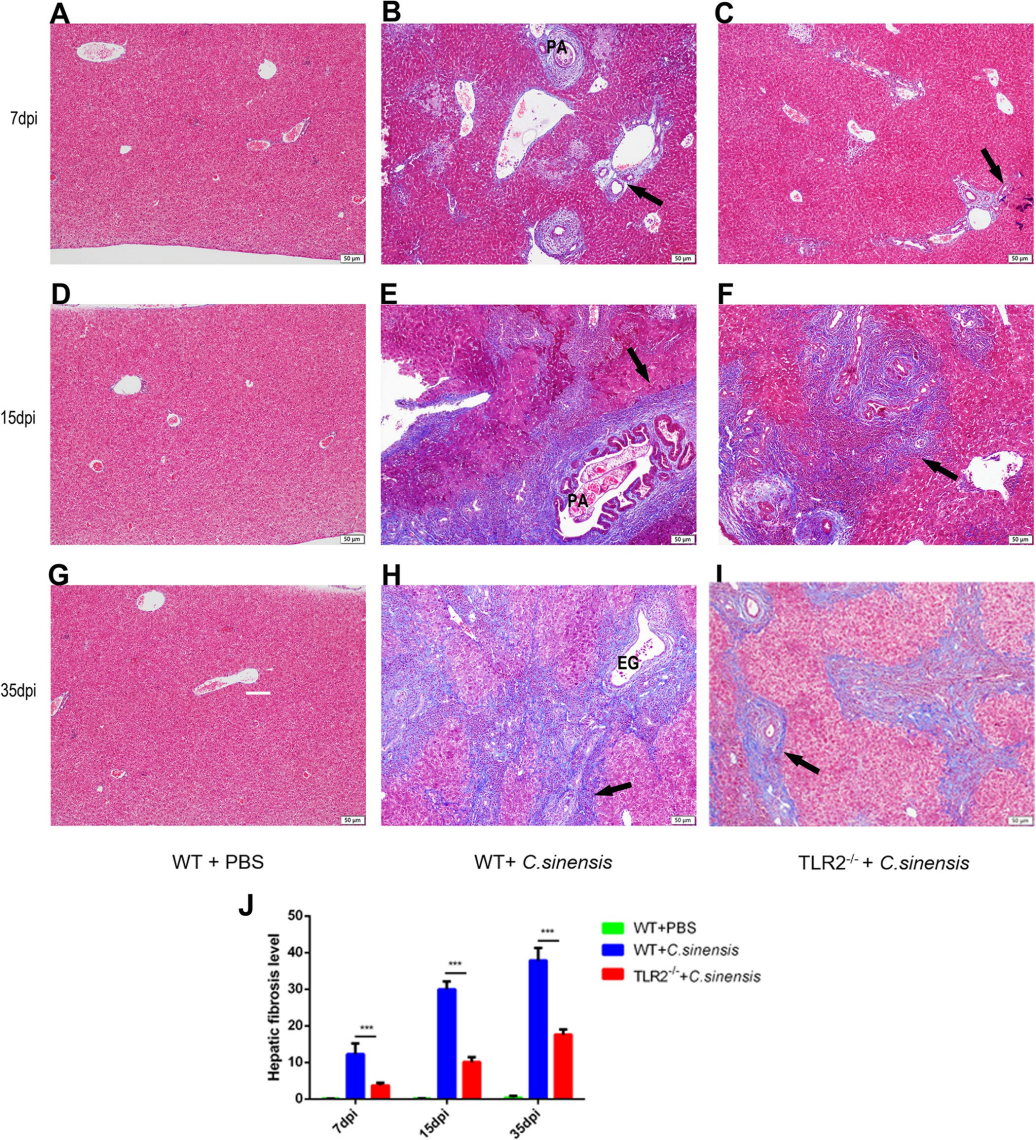
TLR2 deficiency alleviated liver fibrosis in *C*. *sinensis-*infected mice. The WT and *TLR2*^*-/-*^ mice (n = 10) were orally administrated by 200 *C*. *sinensis* metacercariae or PBS, respectively. (A-I) liver fibrosis in *C*. *sinensis*-infected WT and *TLR2*^*-/-*^ mice at 7 dpi, 15 dpi and 35 dpi were visualized by Masson staining. Collagen deposition was visualized by blue stripes and indicated by the black arrows. Eggs were indicated by ‘EG’; Parasites were indicated by the letter ‘PA’. Scale bars = 50 μm. (J) Collagen depositions from each specimen were semi-quantified using Image-Pro Plus software.

We performed Masson staining on the liver tissues of *C*. *sinensis*-infected WT and *TLR2^-/-^* mice to detect collagen deposition, with PBS as the control group. Collagen deposition was visualized by blue stripes in Fig 4A–4I. At 7 dpi, slight collagen deposition was shown around bile duct in *C*. *sinensis*-infected WT mice (Fig 4B), however, almost no collagen deposition was observed around the bile duct of *C*. *sinensis*-infected *TLR2^-/-^* mice (Fig 4C). At 15 dpi, the collagen in liver of *C*. *sinensis*-infected WT mice accumulated around the bile ducts and spread to the hepatic parenchyma (Fig 4E), while a few collagen fibers were found around the bile ducts in *C*. *sinensis*-infected *TLR2^-/-^* mice (Fig 4F). At 35 dpi, the biliary fibrosis of *C*. *sinensis*-infected WT mice continued to deteriorate, which was seen in a large area of fibrosis with bridging fibrosis formed (black arrows indicated) (Fig 4H). The collagen deposition of *C*. *sinensis*-infected *TLR2^-/-^* mice was significantly lower than that in WT mice (Fig 4I). No obvious fibrotic changes were seen in the hepatic tissues of the PBS control mice (Fig 4A and 4D and 4G). Statistical analysis showed that the liver fibrosis grade of *C*. *sinensis*-infected *TLR2^-/-^* mice was significantly lower than that of *C*. *sinensis*-infected WT mice (Fig 4J) (*p*<0.001, *p*<0.001, *p*<0.001). These findings indicated that TLR2 promoted collagen deposition in *C*. *sinensis*-induced liver fibrosis.

### 5. *C*. *sinensis* activated myofibroblasts through TLR2-TGF-β/Smad pathway

**Fig 5:**
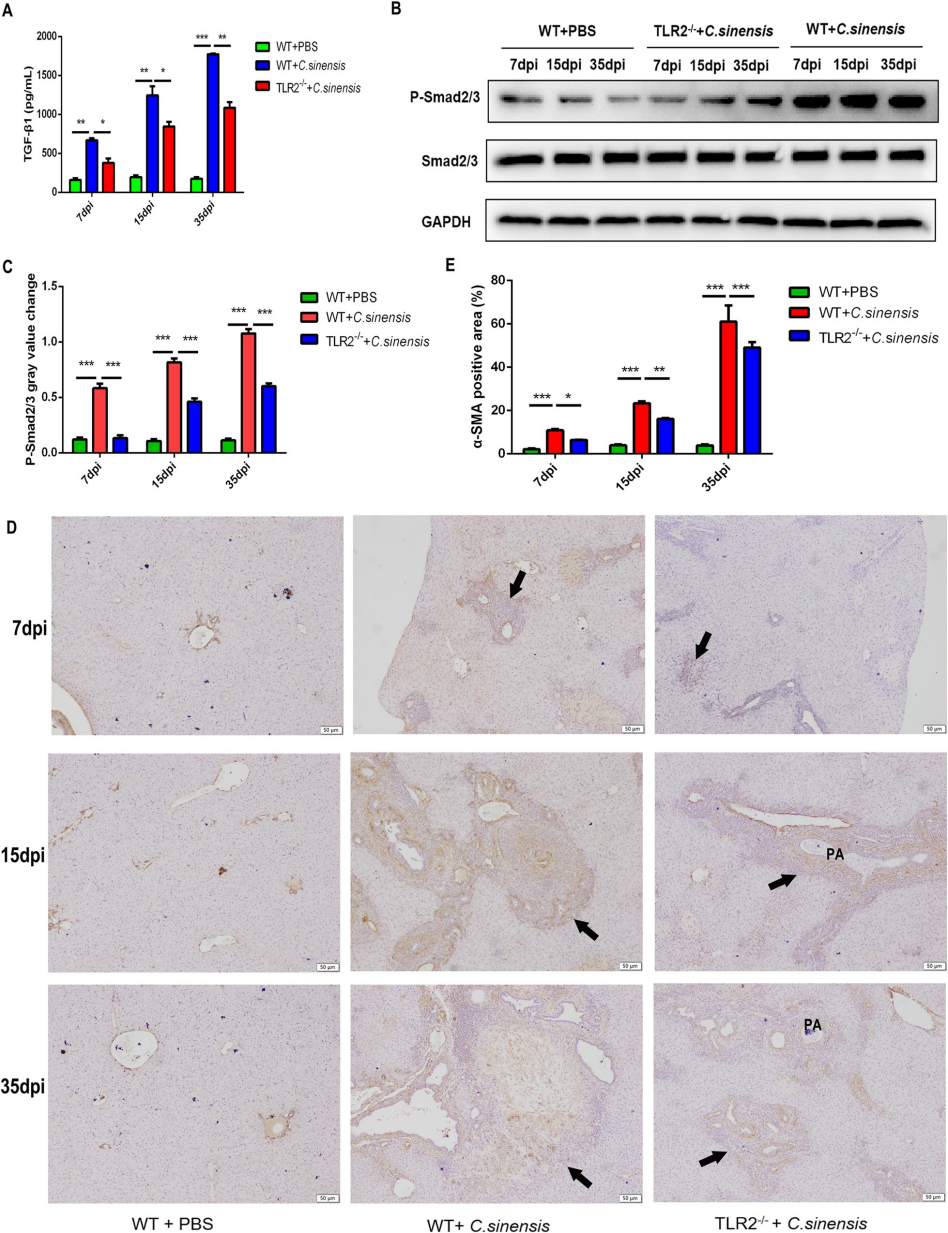
TLR2 deficiency decreased TGF-β1 expression, phosphorylated Smad2/3 and myofibroblasts activation induced by *C*. *sinensis*. The WT and *TLR2*^-/-^ mice (n = 10) were orally administrated by 200 *C*. *sinensis* metacercariae or PBS, respectively. These mice were euthanized and the livers were harvested at 7 dpi, 15 dpi and 35 dpi. (A) The expression level of TGF-β1 was detected by ELISA. (B, C) The phosphorylation of Smad2/3 in liver were analyzed by Western blot. (D) Immunohistochemistry for α-SMA in the liver from WT and *TLR*2^-/-^ mice following 7dpi, 15dpi and 35dpi of *C*. *sinensis* infection. α-SMA positive was shown as yellow and was pointed with black arrows. Scale bar = 50μm. (E) Quantification ofα-SMA positive cells in (D).

The expression level of TGF-β1 increased gradually in the livers of *C*. *sinensis*-infected WT mice, which was higher than that of the mice treated with PBS at 7 dpi, 15 dpi and 35 dpi (Fig 5A) (*p*<0.01, *p*<0.01, *p*<0.0001). TGF-β1 expression in *C*. *sinensis*-infected *TLR2^-/-^* mice liver was partially suppressed at 7 dpi, 15 dpi and 35 dpi compared with that of *C*. *sinensis* infected WT mice (Fig 5A) (*p* <0.05, *p*<0.05, *p* <0.01).

The phosphorylation level of Smad2/3 was significantly up-regulated in *C*. *sinensis*-infected WT mice, compared with that in the mice treated with PBS (Fig 5B and 5C) (*p* <0.001, *p* <0.001, *p* <0.001). The phosphorylated Smad2/3 in *C*. *sinensis*-infected *TLR2^-/-^* mice was significantly decreased compared to that in *C*. *sinensis*-infected WT mice at 7 dpi, 15 dpi and 35 dpi (Fig 5B and 5C) (*p* <0.001, *p* <0.001, *p* <0.001).

Immunohistochemistry of α-SMA (a marker protein for myofibroblasts) were used to determine the location, number of myofibroblasts [21]. Significantly increased expression of α-SMA was found in *C*. *sinensis*-infected WT mice compared to uninfected WT mice at 7 dpi, 15 dpi and 35 dpi (Fig 5D and 5E). The α-SMA proteins distributed around the bile ducts and continued to increase as the duration of infection increased (Fig 5D and 5E) (*p* <0.001, *p* <0.001, *p* <0.001). The number of myofibroblasts was significantly reduced in *TLR2^-/-^* mice compared to WT mice at 7 dpi, 15 dpi and 35 dpi (Fig 5D and 5E) (*p* <0.05, *p* <0.01, *p* <0.001). These results suggested that *C*. *sinensis* increased myofibroblasts activation via TLR2-TGF-β/Smad pathway.

### 6. *C*. *sinensis* promoted the expressions of cytokines in liver and BECs through TLR2 pathway

**Fig 6:**
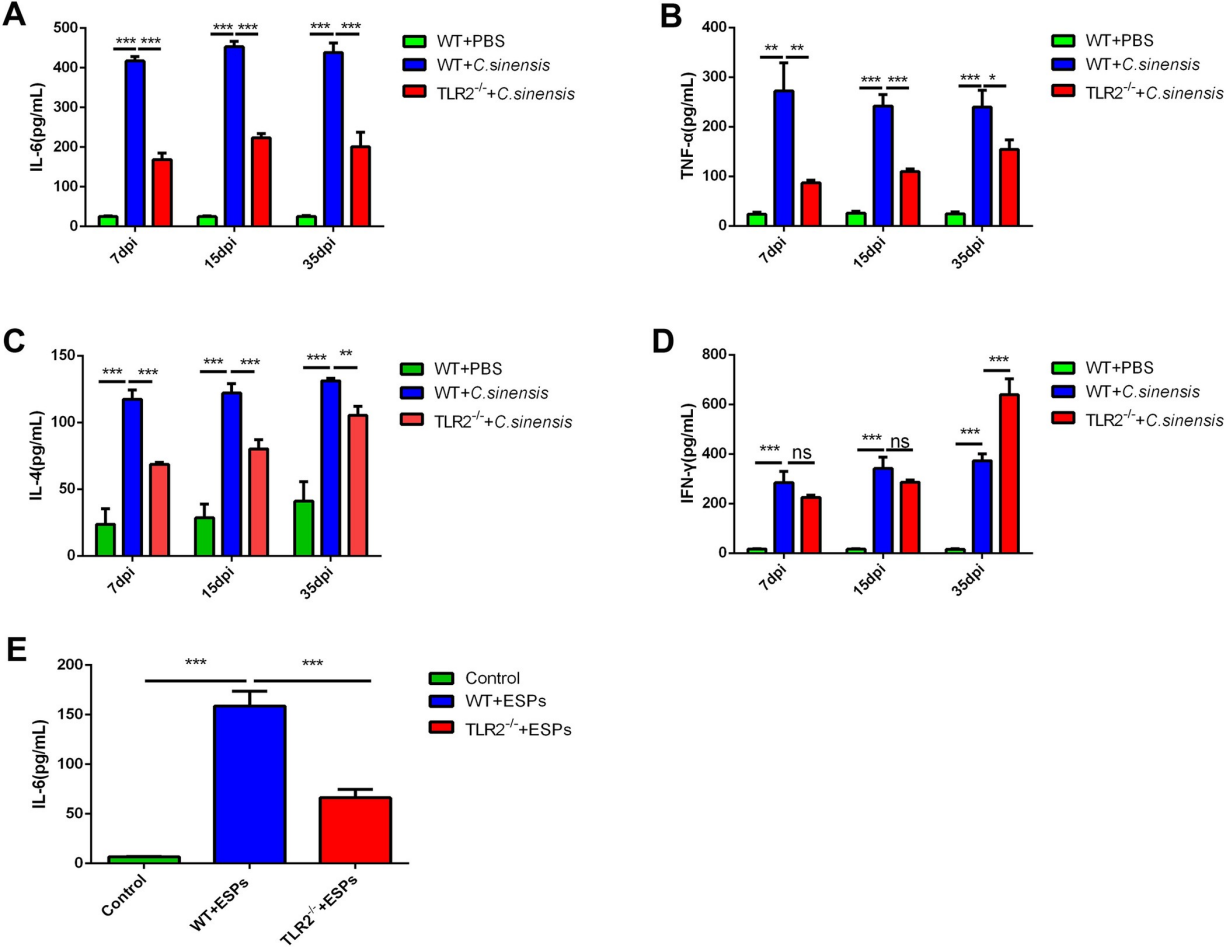
*C*. *sinensis* promoted the expressions of cytokines in liver and BECs through TLR2 pathway. (A-D) The expression of IL-6, TNF-α, IL-4 and IFN-γ in the livers of WT and *TLR2*^*-/-*^ mice were detected separately by ELISA. (E) 3×10^5^ WT mouse BECs were co-cultured with ESPs (50 μg/mL) for 18 h, the cell supernatant was detected by ELISA for IL-6 expression.

We further determined the effect of TLR2 in mediating inflammation in host liver during *C*. *sinensis* infection by ELISA. Our results showed that *C*. *sinensis* significantly increased the expressions of IL-6 (Fig 6A) (*p*<0.001, *p*<0.001, *p*<0.001), TNF-α (Fig 6B) (*p*<0.01, *p*<0.001, *p*<0.001), IL-4 (Fig 6C) (*p*<0.001, *p*<0.001, *p*<0.001) and IFN-γ (Fig 6D) (*p*<0.001, *p*<0.001, *p*<0.001) in WT mice compared to that in PBS treated WT mouse liver. The expression of IL-6 (Fig 6A) (*p*<0.001, *p*<0.001, *p*<0.001), TNF-α (Fig 6B) (*p*<0.01, *p*<0.001, *p*<0.05) and IL-4 (Fig 6C) (*p*<0.001, *p*<0.001, *p*<0.01) in *C*. *sinensis-*infected *TLR2^-/-^* mice were significantly reduced compared to that of *C*. *sinensis*-infected WT mice. In contrast, the expression of IFN-γ was significantly elevated in *TLR2^-/-^* mice compared to that in WT mice at 35 dpi (Fig 6D) (*p*>0.05, *p*>0.05, *p*<0.05).

To further study whether the expressions of cytokines were caused by *C*. *sinensis* ESPs, we examined the cytokines in the culture supernatant of WT mouse BECs stimulated with *C*. *sinensis* ESPs. The secretion level of IL-6 was significantly increased in WT mouse BECs incubated with *C*. *sinensis* ESPs, compared with WT mouse BECs without *C*. *sinensis* ESPs (Fig 6E) (*p*<0.001). The secretion levels of IL-6 were significantly decreased in *TLR2^-/-^* mouse BECs after incubation with *C*. *sinensis* ESPs, compared with WT mouse BECs (Fig 6E) (*p*<0.001). No TNF-α, IL-4 and IFN-γ expression was detected in either WT or *TLR2^-/-^* mouse BECs stimulated with *C*. *sinensis* ESPs. Taken together, these data indicated that *C*. *sinensis* significantly upregulated IL-6 expression in liver and BECs via TLR2.

### 7. *C*. *sinensis* regulated IL-6 production in mice liver and BECs through TLR2-mediated AKT and p38 pathways

**Fig 7:**
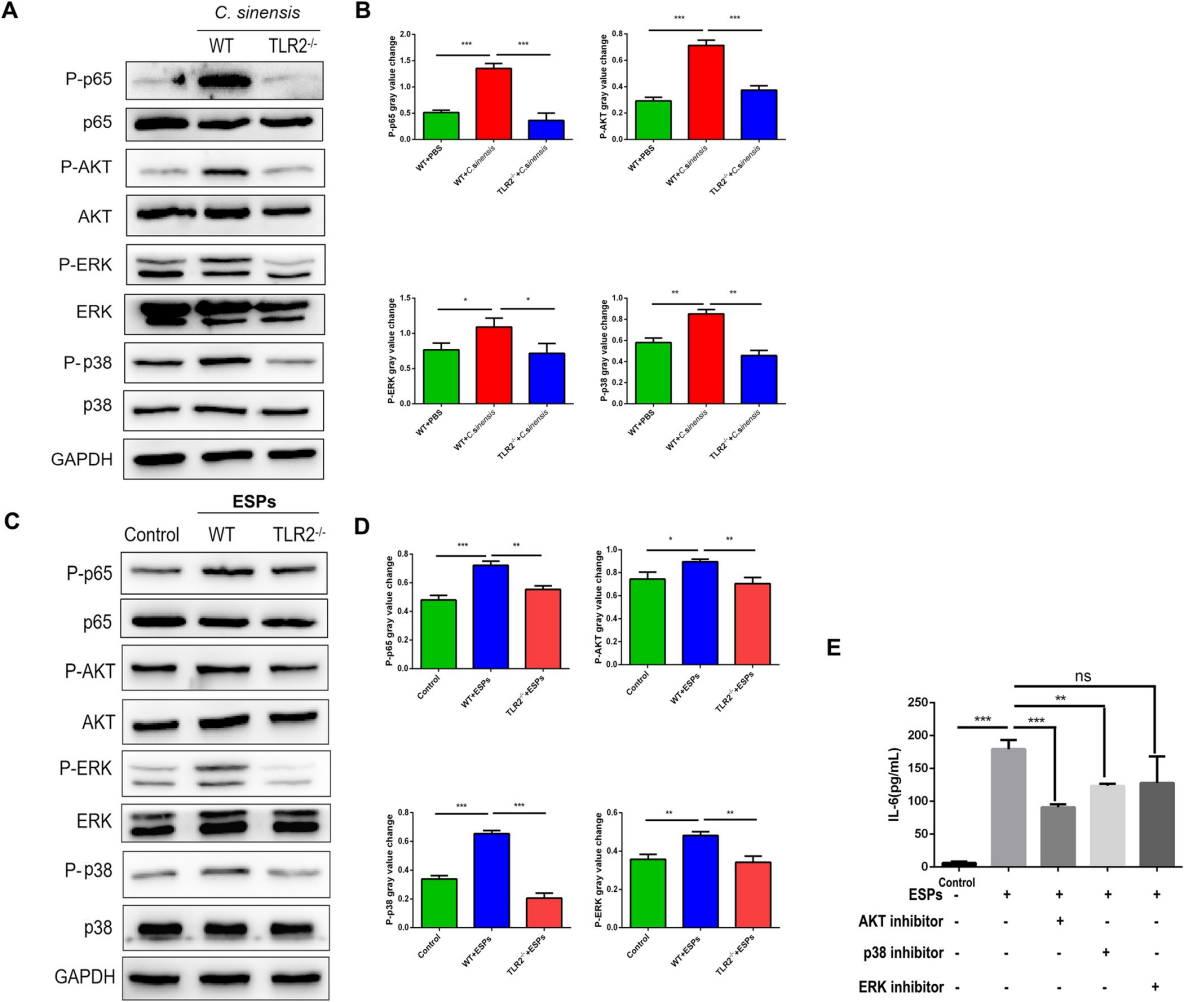
*C*. *sinensis* ESPs increased the production of IL-6 via TLR2-mediated AKT and p38 pathways. (A, B) The phosphorylation of AKT, p65, ERK and p38 in liver were analyzed by Western blot. (C, D) 3 × 10^5^ WT and *TLR2*^*-/-*^ mouse BECs were cultivated with *C*. *sinensis* ESPs (50 μg/mL) for 120 min, the cells were lysed in cells lysates and the phosphorylation of AKT, p65, ERK and p38 were analyzed by Western blot. (E) The production levels of IL-6 in the supernatant of BECs pretreated with or without AKT inhibitor, p38 inhibitor and ERK inhibitor for 1 h, then co-cultured with ESPs for 18 h were measured by ELISA.

The phosphorylation of AKT, p65, ERK and p38 were detected in mice liver and BECs by Western blot. The phosphorylation of AKT, p65, ERK and p38 in liver of WT mice infected with *C*. *sinensis* were significantly increased (Fig 7A and 7B) (*p*<0.001, *p*<0.001, *p*<0.05, *p*<0.01), which were suppressed by TLR2 deficiency (Fig 7A and 7B) (*p*<0.001, *p*<0.001, *p*<0.05, *p*<0.01). WT and *TLR2^-/-^* mouse BECs were treated with *C*. *sinensis* ESPs for 120 min. The phosphorylation of AKT, p65, ERK and p38 were significantly increased in *C*. *sinensis* ESPs treated WT mouse BECs (Fig 7C and 7D) (*p*<0.001, *p*<0.05, *p*<0.001, *p*<0.01) which were significantly reduced in *TLR2^-/-^* mouse BECs compared to WT mouse BECs (Fig 7C and 7D) (*p*<0.01, *p*<0.01, *p*<0.001, *p*<0.01).

To investigate the role of AKT, ERK and p38 pathways in regulating the production of IL-6, we used AKT inhibitor, p38 inhibitor and ERK inhibitor to pretreat WT BECs for 60 min at 37°C, with untreated cells as the control. Subsequently, the treated and untreated cells were incubated with ESPs for 18 h, and the expression of IL-6 in the supernatant were detected by ELISA. The results showed that AKT and p38 inhibitors significantly inhibited IL-6 production (Fig 7E) (*p*<0.01, *p*<0.05). The results indicated that *C*. *sinensis* promoted IL-6 release in mice liver or BECs via TLR2-AKT/P38 pathways.

## Discussion

The *C*. *sinensis* adults usually reside in the peripheral intrahepatic bile ducts, causes persistent injury and repair of intrahepatic bile duct tissue, resulting in intrahepatic duct dilatation and cholangitis, which leads to various complications including gallstones, liver fibrosis and cirrhosis.

More studies on the pathogenesis of clonorchiosis are necessary in mouse models. Previous study have reported that 35 *C*. *sinensis* metacercariae infection caused biliary fibrosis at 84dpi and 112dpi in C3H/HeN mice [9]. The worm recovery rates in *C*. *sinensis*-infected C3H/HeN, C57BL/6, BALB/c, ICR, DDY and CBA/N mice with 150 metacercariae were 20% /0.83%, 9.3%/10%, 16%/8.3%, 0%/5%, 13.35/0.67% and 8.3%/0% at 4 and 8 weeks, respectively [7]. In the present study, we established a *C*. *sinensis*-infected biliary fibrosis model with C57BL/6 mice. And the worm recovery rates were 13.17% and 12.67% in the *C*. *sinensis*-infected WT and *TLR2^-/-^* mice with 200 metacercariae at 35 dpi, respectively. Another study showed that the laboratory infection rate of *C*. *sinensis* were 79.17% and 12.50% in BALB/c and C57BL/6 innoculated with 30 metacercariae, respectively [22]. In our research, it was shown that 200 *C*. *sinensis* metacercariae innoculation led to 100% infection, caused liver tissue inflammation and biliary injury, which induced myofibroblasts activation and excessive ECMs deposition around the bile duct to form biliary fibrosis in mice. The establishment of a new mouse model helps to better simulate the pathogenic process of clonorchiosis.

Infections with different numbers of metacercariae in mouse model lead to the development of acute and chronic courses in clonorchiosis. The liver fibrosis and the inflammatory in the present study were more intense than that in the *C*. *siensis*-infected C57BL/6 mouse model with 30 metacercariae describe previously [7]. This mouse model was used as a useful approach to study potential mechanisms of fibrosis, and may shed light on the processes involved in the actual host (dog, cat, human) of the parasite, such as simulating the course of an acute, heavy infection [23]. However, it is important to note that the biliary fibrosis caused by this acute infection (with high fatality within a very short time) may be quite different from that caused by persistent, asymptomatic infection with *C*. *sinensis*. The latter is usually associated with persistent chronic inflammation and has few clinical symptoms [1]. The availability of a TLR^-/-^ mutant C57BL/6 mice makes this model a practical tool to study parasitic liver fibrosis.

TLR2 is involved in the development of *C*. *sinensis*-induced liver fibrosis. Previous study showed that TLR2 deficiency reduces liver inflammation and fibrosis in a mouse model of choline-deficient, L-amino acid-defined (CDAA)-caused liver fibrosis [24]. Bacterial translocation and intestinal inflammation in mice promote hepatic fibrosis though TLR2 signaling pathway in lamina propria mononuclear cells and TNFRI signaling on intestinal epithelial cells [25]. Our data suggested that *C*. *sinensis* induced significantly increased expression of TLR2 in mouse BECs in *vivo* and in *vitro* assays. TLR2 deficiency reduced liver lesions and inflammation, alleviated the liver fibrosis induced by *C*. *sinensis*. Also, *C*. *sinensis*-infected *TLR2^-/-^* mice showed decreased mortality rate. The number of adult worms was significantly lower in *C*. *sinensis*-infected *TLR2^-/-^* mice then that in WT mice. This may be due to the altered immune response induced by *C*. *sinensis* infection in *TLR2^-/-^* mice, which limits the growth and sexual maturation of worms. But how TLR2 regulates the development of *C*. *sinensis* needs to be further explored in future studies.

A question to ponder is that TLR2 and TLR4 appear to be a synergistic promoter of *C*. *sinensis*-induced liver fibrosis. We confirmed that TLR2 promoted the process of parasitic liver fibrosis. TLR2 deficiency resulted in milder liver fibrosis. The same consequence of TLR4 was confirmed in liver fibrosis caused by *C*. *sinensis* [6]. TLR4 deficiency reduces *C*. *sinensis* induced-liver fibrosis by decreasing the expression of key factors TGF-β1 and phospho-Smad2/3 [6].We hypothesized that the functions of TLR2 and TLR4 were relevant and both were required for the host inflammatory regulation process, thus affecting the liver injury and biliary fibrosis induced by *C*. *sinensis* when either of the TLR absent. Studies suggested that both TLR2 and TLR4 are activated in *C*. *sinensis* infected mice maybe supporting this conjecture [9]. In clonorchiosis, deletion of either TLR2 or TLR4 alone significantly downregulated biliary fibrosis caused by *C*. *sinensis*. It provides new information for the clinical understanding of the mechanism of liver fibrosis caused by *C*. *sinensis*.

IL-6, TNF-α and IL-4 are important cytokines involved in liver inflammation, liver injury and liver fibrosis [26–28]. And IFN-γ has been found to promote HSCs apoptosis and to reduce liver fibrosis [29]. Previous studies found that *C*. *sinensis* infection significantly promoted IL-6, TNF-α and IL-4 productions [18,26]. Exocrine components of *C*. *sinensis* can promote the high expression of IL-6 and TNF-α in mouse macrophages [30]. In the present study, the productions of IL-6, TNF-α and IL-4 were significantly elevated in *C*. *sinensis*-induced liver fibrosis, which is consistent with previous studies [18,26]. It is worth mentioning that TLR2 deficiency significantly reduced the productions of IL-6, TNF-α and IL-4, while promoted IFN-γ production, the severity of liver fibrosis was reduced, suggesting that IL-6, TNF-α, IL-4 and IFN-γ were regulated by TLR2 and could be involved in the pathogenesis of biliary fibrosis induced by *C*. *sinensis*. *C*. *sinensis* infection activates TLR2 and TLR4 signals, and induced high expression of TNF-α, IL-10, IL-4, and IFN-γ [9]. And the levels of IFN-γ, IL-6, TNF-α and IL-10 in splenocytes of TLR4-deficient mice were significantly lower than those of WT mice [31]. It was suggested that both TLR2 and TLR4 could regulate the cytokine release in mice induced by *C*. *sinensis* infection. In addition, other liver flukes infection, such as *Opisthorchis viverrini*, also result in high expression of IL-6 and TNF-α, and is closely associated with the development of liver fibrosis [32,33]. Whether the synergistic regulation of TLR2 and TLR4 is also applicable to the pathogenic process of *O*. *viverrini* deserves further investigation in future studies, which may provide more possibilities to unravel liver fibrosis in liver flukes.

TLR2-regulated inflammatory factor expression in *C*. *sinensis*-induced liver fibrosis via the MAPK/ AKT pathways. MAPK, AKT and NF-қB pathways are the main inflammatory signaling pathways involved in the regulation of liver fibrosis [28]. *C*. *sinensis* ESPs CsMF6p/HDM and phospholipase A2 could regulate JNK and p38 to induce pro-inflammatory immune response of macrophages and to activate myofibroblasts [30,34]. In addition, the role of AKT and MAPK pathways in promoting BECs proliferation had been reported [35,36]. We demonstrated that *C*. *sinensis* regulated the phosphorylation of AKT, p65, p38 and ERK via TLR2, this further demonstrated the role of TLR2 in modulating multiple pathways.

**Fig 8:**
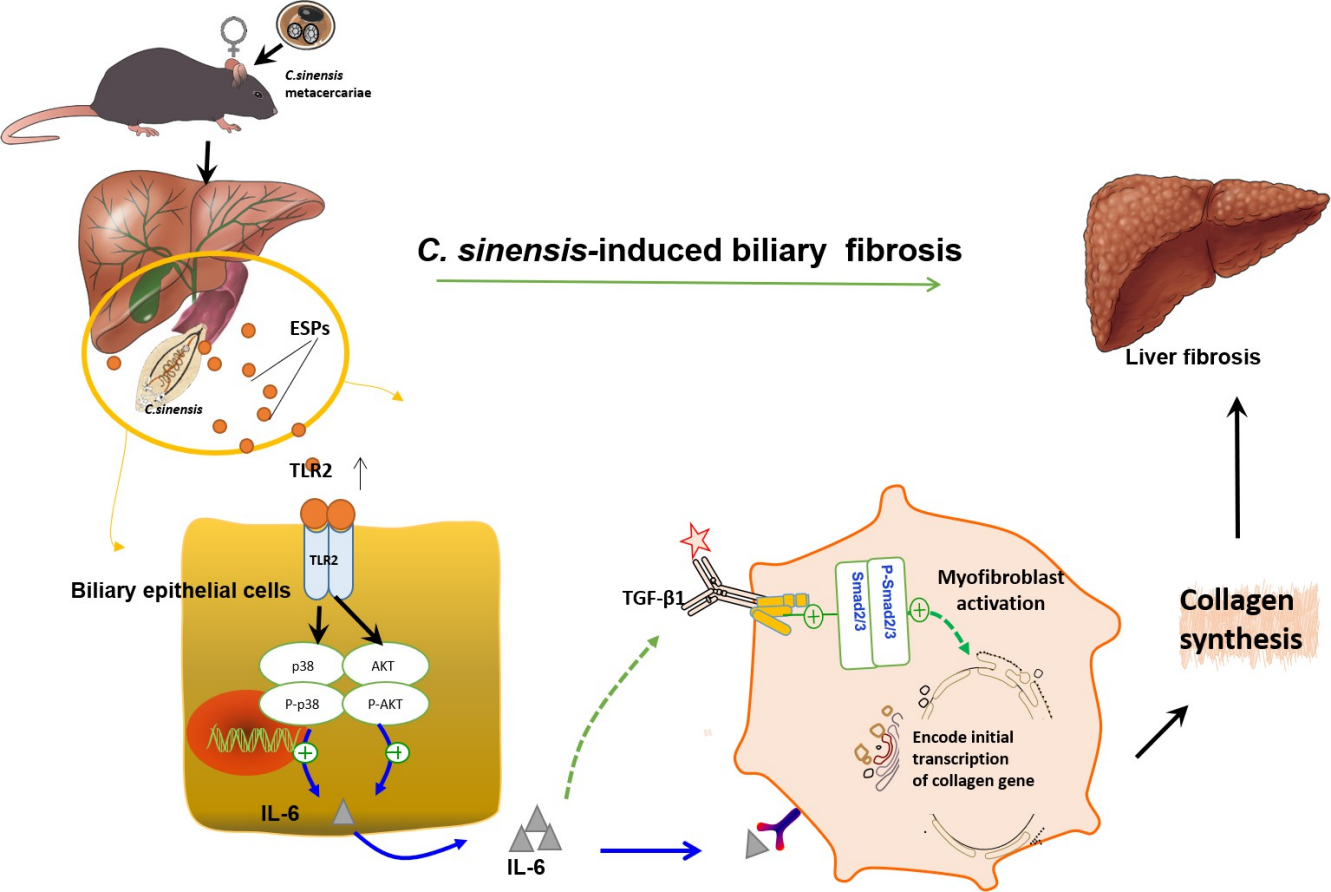
*C*. *sinensis* activated TLR2 signal pathway to aggravate biliary fibrosis through promoting IL-6 production. *C*. *sinensis* triggered AKT and p38 signal pathways dependent on TLR2 of BECs to promote the production of IL-6, which subsequently activated TGF-β1-Smad2/Smad3 pathways, myofibroblasts and up-regulated the expression of ECMs, finally resulting in biliary fibrosis in mice. In the figure, ‘**↑**’indicates positive regulation.

In summary, a new role of TLR2 in aggravating *C*. *sinensis*-induced parasitic liver fibrosis was identified. TLR2 triggered TGF-β1/Smad2/3 and p38/AKT signaling pathways played crucial roles in activating myofibroblasts and promoting the production of IL-6, which exacerbated liver fibrosis caused by *C*. *sinensis* (Fig 8). This study provides a useful mouse model to study the potential mechanisms of parasite-induced liver fibrosis, and explores the role of TLR2 deficiency in liver fibrosis, providing important reference information for understanding liver fibrosis caused by *C*. *sinensis*.

## Supporting information

The numerical data used in all figures are included in S1 Data.

S1 Data Excel spreadsheet containing, in separate sheets, the underlying numerical data and statistical analysis for Figure panels 1A, 1B, 1L, 1M, 2A-C, 3D, 4J, 5A, 5C, 5E, 6A-E, 7B, 7D and 7E.
(XLSX)
